# PathCards: multi-source consolidation of human biological pathways

**DOI:** 10.1093/database/bav006

**Published:** 2015-02-27

**Authors:** Frida Belinky, Noam Nativ, Gil Stelzer, Shahar Zimmerman, Tsippi Iny Stein, Marilyn Safran, Doron Lancet

**Affiliations:** Department of Molecular Genetics, Weizmann Institute of Science, Rehovot 7610001, Israel

## Abstract

The study of biological pathways is key to a large number of systems analyses. However, many relevant tools consider a limited number of pathway sources, missing out on many genes and gene-to-gene connections. Simply pooling several pathways sources would result in redundancy and the lack of systematic pathway interrelations. To address this, we exercised a combination of hierarchical clustering and nearest neighbor graph representation, with judiciously selected cutoff values, thereby consolidating 3215 human pathways from 12 sources into a set of 1073 SuperPaths. Our unification algorithm finds a balance between reducing redundancy and optimizing the level of pathway-related informativeness for individual genes. We show a substantial enhancement of the SuperPaths’ capacity to infer gene-to-gene relationships when compared with individual pathway sources, separately or taken together. Further, we demonstrate that the chosen 12 sources entail nearly exhaustive gene coverage. The computed SuperPaths are presented in a new online database, PathCards, showing each SuperPath, its constituent network of pathways, and its contained genes. This provides researchers with a rich, searchable systems analysis resource.**Database URL:** http://pathcards.genecards.org/

## Introduction

**Figure 1.**
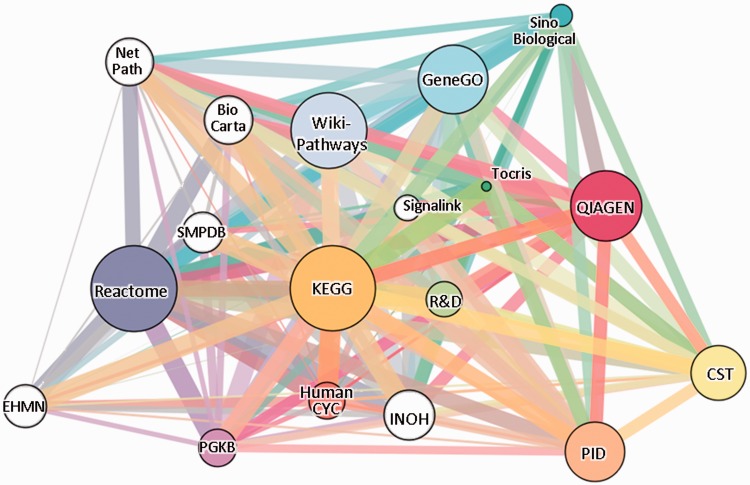
The gene-content network of pathway sources. Eighteen sources are shown, 12 of which (colored) are included in SuperPaths generation. Edge widths are proportional to the pairwise Jaccard similarity coefficient computed for the gene contents of the entire source. The sources, depicted in GeneCards Version 3.12, are: Reactome (13), KEGG (14), PharmGKB (15), WikiPathways (16), QIAGEN, HumanCyc (17), Pathway Interaction Database (18), Tocris Bioscience, GeneGO, Cell Signaling Technologies (CST), R&D Systems and Sino Biological (see Table 1). White circles correspond to sources not included in the SuperPath generation process: BioCarta (19), SMPDB (20), INOH (21), NetPath (22), EHMN (23) and SignaLink (24).

The systematic analysis of biological pathways has ever-increasing significance in an age of growing systems analyses and omics data. Mapping genes onto pathways may contribute to a better understanding of biological and biomedical mechanisms. The literature provides a large collection of pathway definition sources (1). Pathway knowledge bases represent the careful collection of genes and their interactions, mapped onto biological processes. These repositories, which include both academic and commercial resources (Figure 1A), provide lists of pathways and their cellular components, each with an idiosyncratic view of the pathway universe.

Indeed, the definition of the boundaries of biological pathways differs among sources, as exemplified by the highly studied processes of fatty acid metabolism (2) or the TCA cycle (the tricarboxylic acid cycle) (3). Further, the same pathway name may have widely dissimilar gene content in different sources (4). At present, there is no definitive analysis of pathway similarities, either between or within sources. Thus the multitude of pathway resources can often be confusing when portraying gene-pathway affiliations.

Previous attempts to unify pathways from several sources include NCBI’s Biosystems (5), PathwayCommons (6), PathJam (7), HPD (8), ConsensusPathDB (9), hiPathDB (10) and Pathway Distiller (11). But none of these efforts entail a standardized method to unify numerous sources into a consolidated global repository.

Here, we describe an approach aimed at generating an integrated view across multiple pathway sources. We applied a combination of nearest neighbor graph and hierarchical clustering, utilizing a gene-content metric, to generate a manageable set of 1073 unified pathways (SuperPaths). These optimally encompass all of the information contained in the individual sources, striving to minimize pathway redundancy while maximizing gene-related pathway informativeness. The resultant SuperPaths are integrated into GeneCards (12), enabling clear portrayal of a gene’s set of unified pathways. Finally, these SuperPaths, together with diverse related biological data, are provided in PathCards—a new pathway-centric online database, enabling quick in-depth analysis of each human SuperPath.

## Materials and methods

### Pathway mining and comparison

Pathway gene sets were generated based on the GeneCards platform (12), implementing the gene symbolization process allowing for comparison of pathway gene sets, from 12 different manually curated sources, including: Reactome (13), KEGG (14), PharmGKB (15), WikiPathways (16) QIAGEN, HumanCyc (17), Pathway Interaction Database (18), Tocris Bioscience, GeneGO, Cell Signaling Technologies (CST), R&D Systems and Sino Biological (see Table 1). A binary matrix was generated for all 3125 pathways, where each column represents a gene indicated by 1 for presence in the pathway and 0 for absence. Additionally, six sources were analysed for their cumulative tallying of genes content, including: BioCarta (19), SMPDB (20), INOH (21), NetPath (22), EHMN (23) and SignaLink (24).

**Table 1.** Pathway sources

Source	Number of pathways	Number of genes	Pathway size average	Pathwaysize stdev	% of singletons	Reference
Reactome	1411	7157	46.2	105.5	2.5	13
KEGG	284	6746	81.9	91.2	28.8	14
QIAGEN	317	3626	123.1	124.2	17.6	http://www.qiagen.com/geneglobe/
HumanCyc	319	831	6.5	7.6	10.0	17
GeneGO	250	3413	48.7	22.1	22.8	http://lsresearch.thomsonreuters.com/maps/
WikiPathways	229	4504	48.1	46.0	41.5	16
Pathway Interaction Database	186	2239	34.9	21.1	62.9	18
PharmGKB	102	2239	16.4	14.5	29.4	15
RnD systems	36	863	52.1	28.6	22.2	http://www.rndsystems.com/Pathways.aspx
Cell signaling technologies	21	1820	127.4	63.4	80.1	http://www.cellsignal.com/contents/science/cst-pathways/science-pathways
Tocris	12	263	55.6	29.2	8.3	http://www.tocris.com/signalling Path ways. php
Sino Biological	11	450	64.9	34.9	27.3	http://www.sinobiological.com/

### Pathway similarity assessment

In the analyses performed, we utilized gene content overlap to estimate pathway similarity. This was done based on the Jaccard coefficient, that measures similarity between finite sample sets, and defined as the size of the intersection divided by the size of the union of the sets. To examine the legitimacy of this method, we performed a comparison to an alternative methodology, embodied in MetaPathwayHunter pathway comparison, that incorporates topology in pairwise pathway alignment (25). For such analysis, we used a set of 151 yeast pathways available in MetaPathwayHunter, and computed Jaccard similarity coefficients (*J*) for all 11 325 pathway pairs. We then selected a sample of 30 pairs containing 28 unique pathways out of a total of 87 pairs with *J* ≥ 0.3, ensuring maximal representation for larger pathways. Each of the 28 pathways was queried in MetaPathwayHunter against the entire gamut of 151 with default parameters (a total of 4228 comparisons). We found that 29 out of the 30 sample pathway pairs obtained a significant MetaPathwayHunter alignment (*P* ≤ 0.01). As only 64 of the 4228 comparisons showed such a *P*-value, the probability of obtaining this result at random is 1.6 × 10^−^^53^ (Supplementary Table S1). Thus, Jaccard scores appear as excellent predictors for the results of the more elaborate method. A full account of interpathway pairwise similarity is available upon request.

### Clustering algorithm

For the main pathway clustering algorithm, we applied a method described elsewhere (26), which includes the following steps: i) The generation of cluster cores by joining all pathway pairs with Jaccard coefficient ≥*T_2_*, the upper cutoff, equivalent to hierarchical clustering. ii) Performing cluster extension by generating new best edges, i.e. joining every pathway to a pathway showing the highest score, as long as it is ≥*T_1_*, the lower cutoff, akin to nearest neighbor joining. If two or more target pathways have the same best score, all are joined. Each resultant connected component is defined to be a pathway cluster (SuperPath). Identical pathway sets were joined without considering each other as nearest neighbors (i.e. the best scoring non-identical pathway gene-set is chosen as the nearest neighbor). This clustering algorithm is order independent.

### Determination of cutoffs

Uniqueness of a SuperPath $U_{s}$ is defined as $\log_{10}\left(\frac{\sum \frac{1}{N_{\text{p}}}}{N_{\text{g}}}\right)$ where *N*_p_ is the number of pathways that include a certain gene, averaging for each pathway over all genes in the SuperPath (divided by the number of genes *N*_g_). Uniqueness of genes $I_{s}$is symmetrically defined per SuperPath as $\log_{10}\left(\frac{\sum \frac{1}{N_{\text{g}}}}{N_{\text{p}}}\right)$ where each *N*_g_ is the number of genes included in the relevant pathway, averaging for each gene over all SuperPaths including a gene. In order to then find the best tradeoff between the two scores, we summed up the average *U_s_* and *I_s_* for each set of *T_1_* and *T_2_* cutoff parameters. Thus *U_s_ + I_s_* was calculated for each set of parameters to find the two parameters for which the tradeoff between pathway and gene uniqueness would be optimal. The best cutoffs by maximizing *U_s_* + *I_s_* were *T_1_* = 0.3 and *T_2_* ≥ 0.5. Further fine tuning of the upper cutoff was performed by resampling of the data, a technique employed by Levin and Domany (27). We used two dilutions (0.75 and 0.9), i.e. randomly sampling 75% and 90% of the pathways (resampling 100 times for each dilution) and performing the clustering algorithm on each sample, each time calculating the percent of the edges present in the original clustering—the percent of cases that two pathways belonged to the same cluster as in the full dataset. In both dilutions, the upper cutoff of 0.7 was found to recover a higher percent of the edges in the original clustering algorithm (Figure 4C).

### Name similarity calculation and concordance with gene similarity

Name similarity was calculated as the Jaccard coefficients of the shared words in the two pathway names, after omitting trivial words and using stemming to identify words with the same root. The cutoff between similar and non-similar names (as well as gene content in regard to comparison with name similarity) was set to *J* = 0.5. Name similarity was compared with gene content similarity to find the level of concordance between the two.

### Shared publications and PPI data

Publication and Protein-Protein Interactions (PPI) data for each gene were obtained from the GeneCards database, including several combined sources. Publications sources of GeneCards include both manually curated publications (e.g. UniProtKB/Swiss-Prot) as well as text mining approaches that report connections between a gene and a list of publications. A shared publication between two genes is an association of both genes to the same publication and does not indicate a direct interaction between the genes. PPI scores between pairs of genes are also based on several interaction sources in GeneCards. Unlike shared publications, PPIs reflect direct interactions between the two gene products.

### Randomization and comparison

A randomized set of pseudo-SuperPaths was generated, such that the pseudo-SuperPaths are the same size and quantity as the SuperPaths, albeit with genes assigned at random (from the list of genes with any pathway annotation). Gene pairs that belong to at least one SuperPath, but do not belong together in any individual pathway (the test set) were analysed for the number of shared publications and PPI scores for each pair. In comparison, gene pairs that belong to at least one pseudo-SuperPath, but do not belong together in any individual pathway (the control set) were analysed for the same attributes. To compare the two sets which are of different sizes, a random sample of the larger set (the control set) of the same size as the smaller set (the test set) was compared with the smaller set. A one-sided Kolmogorov–Smirnoff test was performed to compare between the test and control sets.

### Gene enrichment analysis comparison

Differentially expressed sets of genes were obtained from the GeneCards database (12) containing 830 different embryonic tissues based on manual curation (28). For the comparison of SuperPaths and their pathway constituents, 89 SuperPaths that contained exactly two pathways with Jaccard similarity coefficient <0.6 were chosen, a value selected to include pairs of relatively dissimilar pathways in order to enhance comparative power. Two gene set enrichment analyses were run for all 830 gene sets: one with SuperPaths and the other with their constituent pathways. Whenever both SuperPath and the constituent pathways received a statistical enrichment score, the difference between negative log *P* values was computed.

### GeneCards and PathCards

SuperPaths have been implemented in GeneCards and are now included in the standard procedure of GeneCards generation. PathCards is an online compendium of human pathways, based on the GeneCards database, presenting SuperPath-related data in each page.

## Results

### Pathway sources

We analysed 12 pathway sources included in GeneCards http://www.genecards.org/ (12) with a total of 3215 biological pathways (Table 1 and Figure 1A). The total number of genes covered by these sources is 11 478, nearly twice as large as the gene count in the largest source (Figure 1B), suggesting the power of analysing multiple sources. Asymptotic behavior is observed in the change of total gene count with increasing number of sources. When considering the incorporation of six additional sources (Supplementary Figure S1), we found that the gene count increment is ∼2% of the currently analysed total. This is an indication that the chosen 12 sources provide adequate coverage of human gene-pathway mappings. Switching between the six non-included sources and six included sources of similar size give a very similar graph, with merely 4% increment in gene count (Supplementary Figure S1).

**Figure 2.**
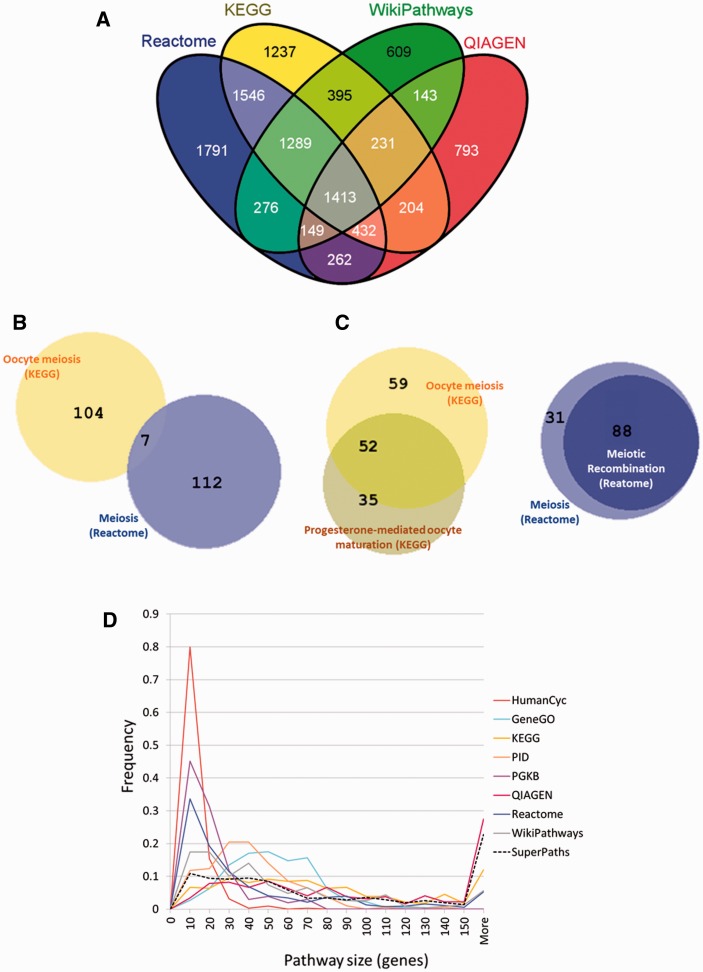
Discrepancies between pathway sources. **(A)** Incomplete gene overlap among sources. Venn diagram (created using VENNY http://bioinfogp.cnb.csic.es/tools/venny/) showing the number of shared genes among the four largest pathway sources. For a total of 10 770 genes, only 1413 (13%) are shared by all four sources and 609–1791 genes are unique to each of these sources. **(B)** Inconsistency of names *versus* content in meiosis-related pathways. A Venn diagram created using BioVenn (29), exemplifies two pathways, ‘Meiosis’ from Reactome and ‘Oocyte meiosis’ from KEGG with very small gene sharing (7 genes out of 172, *J* = 0.04). **(C)** Redundancy in meiosis-related pathways. This is exemplified by the large number of genes (88 of 119, *J* = 0.74) shared by ‘Meiosis’ and ‘Meiotic recombination’ pathways both from Reactome, and by the large number of genes (52 of 146, *J* = 0.36) shared by ‘Oocyte meiosis’ and ‘Progesterone-mediated oocyte maturation’ both from KEGG. **(D)** Pathway size distribution across sources. The pathway size in gene count, is distributed differently across the different sources.

**Figure 3.**
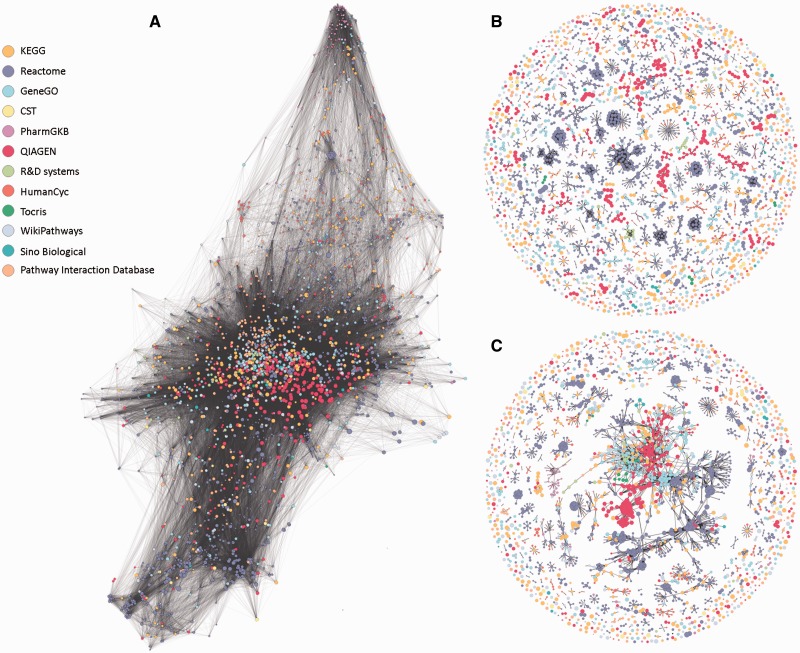
Network representations of the 3215 analyzed pathways. Nodes represent pathways and edges represent Jaccard similarity coefficients (*J*) using different methods. Network visualizations were performed using Gephi (30). Colors correspond to pathway sources. **(A)** No clustering. All edges with *J* ≥ 0.05 are shown. All but 20 pathways form one large connected component with an average degree of 134. **(B)** SuperPaths. Each is a connected component obtain by the main clustering algorithm, with thresholds *T_1_* (best edges) of *J* ≥ 0.3 and *T_2_* of *J* ≥ 0.7. There are 544 singletons and 529 multi-pathway clusters; the size of the largest cluster is 70. **(C)** Pure hierarchical clustering, with thresholds *T_2_* of *J* ≥ 0.3. There are 544 singletons and 288 multimembered clusters; the size of the largest cluster is 1046 pathways.

Analysing the gene repertoires of the four largest sources (Figure 2A), we found that among the 10 770 genes contained within these sources, only 1413 genes were jointly covered by all four sources, and that more than 4000 were unique to one of the four sources. This highlights the notion that source unification is essential to obtain maximal gene coverage. In its simplest embodiment, source unification would entail presenting a unified list of the 3215 pathways included in all 12 sources. This however would ignore the extensive gene-content connectivity embodied in the network representation of this pathway collection (Figure 3A). Further, the original pathway collection has considerable inconsistencies of relations between pathway name and pathway gene content, as exemplified in Figure 2B and C. The summary in Table 2A suggests that only ∼9.4% of all pathway pairs with a similar name have similar gene content, and likewise, only 9.8% of all pathway pairs with similar gene content are named similarly (Supplementary Figure S2).

**Figure 4.**
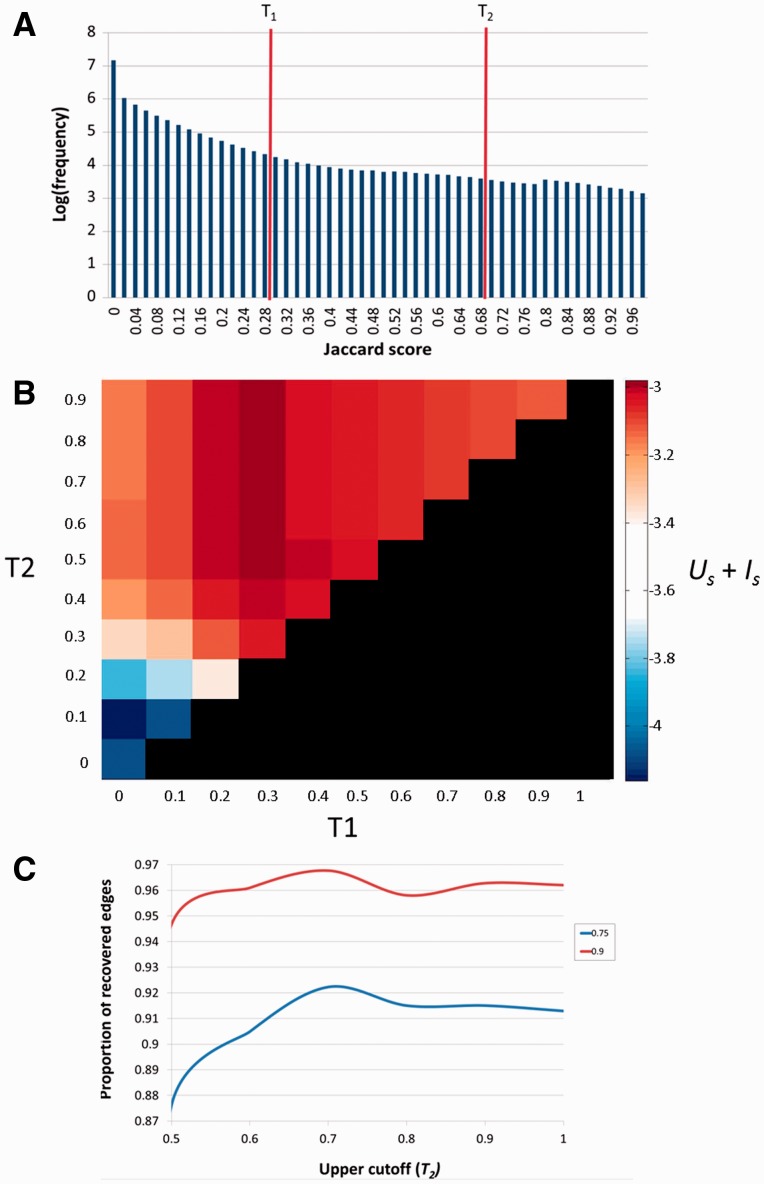
Selection of the *T_1_* and *T*_2_ thresholds. **(A)** Distribution of Jaccard coefficients across all pathway pairs. *T_1_* and *T*_2_ respectively represent the lower and upper cutoffs used in the algorithm employed. **(B)** *U_s_* + *I_s_* scores across combinations of *T_1_* and *T_2_*. The diagonal (*T_1_* = *T_2_*) represents pure hierarchical clustering with different thresholds. The best scores are attained when *T_1_* = 0.3 and *T_2_* ≥ 0.5. **(C)** Determination of *T_2_*. *T_2_* (upper cutoff) was determined by resampling of the pathway data at two dilution levels (27), 0.75 and 0.9. In both cases *J* = 0.7 was found to be the optimum in which a higher fraction of the original clustering is recovered.

**Table 2.** Gene content *versus* name similarity of pathways and SuperPaths

	Non-similar genes	Similar genes
A Pathways		
Similar name	3991	414
Non-similar name	5.15 × 10^6^	3782
B SuperPaths		
Similar name	668	0
Non-similar name	5.74 × 10^5^	3

### Pathway clustering

We performed global pathway analysis aimed at assigning maximally informative pathway-related annotation to every human gene. For this, we converted the pathway compendium into a set of connected components (SuperPaths), each being a limited-size cluster of pathways. We aimed at controlling the size of the resulting SuperPaths, so as to maintain a high measure of annotation specificity and minimize redundancy.

The following two steps were used in the clustering procedure, in which pathways were connected to each other to form SuperPaths. i) Preprocessing of very small pathways: pathways smaller than 20 genes were connected to larger pathways (<200 genes) with a content similarity metric of ≥0.9 relative to the smaller partner. ii) The main pathway clustering algorithm: this was performed using the Jaccard similarity coefficient (*J*) metric (31) (see Materials and Methods). We used a combination (cf. 26) of modified nearest neighbor graph generation with a threshold *T_1_* and hierarchical clustering with a threshold *T_2_* (Figure 4A and Materials and Methods).

To determine the optimal values of the thresholds *T_1_* and *T_2_*, we defined two quantitative attributes of the clustering process. The first is *U_S_*, the overall uniqueness of the set of SuperPaths. *U_S_* elevation is the result of increasing pathway clustering, and reflects the gradual disappearance of redundancy, i.e. of cases in which certain gene sets are portrayed in multiple SuperPaths. The second parameter is *I_S_*, the overall informativeness of the set of SuperPaths. *I_S_* is a measure of how revealing a collection of SuperPaths is for annotating individual genes. It decreases with the extent of pathway clustering, reaching an undesirable minimum of one exceedingly large cluster, whereby identical SuperPath annotation is obtained for all genes. We thus sought an optimal degree of clustering whereby *U_S_ + I_S_* is maximized (Figure 4B and Materials and Methods).

**Figure 5.**
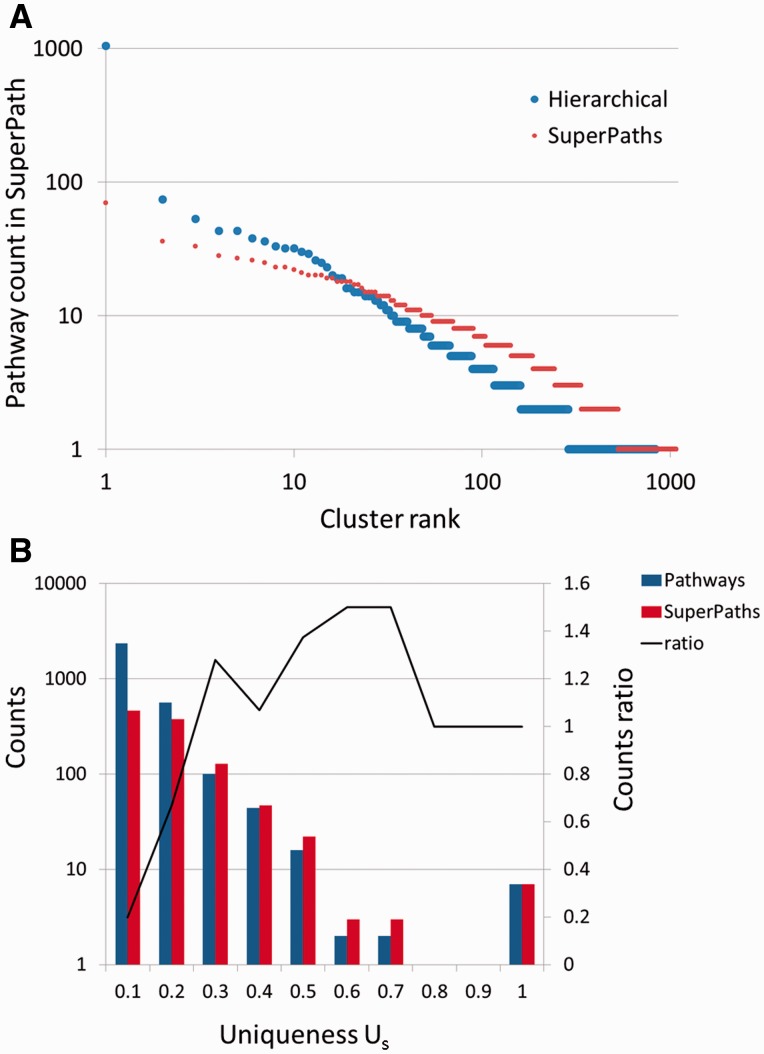
SuperPaths increase uniqueness while keeping high informativeness. (**A**) Number of pathways in hierarchical clustering *versus* SuperPath algorithm. The largest cluster with hierarchical clustering includes 1046 pathways, about 33% of the entire input, causing a great reduction of informativeness. In the SuperPath clustering the maximum cluster size is 70, about 2% of all pathways. (**B**) Increase in uniqueness (*U_s_*) following unification of pathways into SuperPaths.

Our procedure pointed to an optimum at *T_1_* = 0.3 and *T_2_* ≥ 0.5. Further fine tuning by data resampling suggested an optimal value of *T_2_* = 0.7 (Figure 4C and Materials and Methods). This procedure resulted in the definition of 1073 SuperPaths, including 529 SuperPaths ranging in size from 2 to 70 pathways, and 544 singletons (one pathway per SuperPath) (Figures 3B and 5A). Each SuperPath had 3 ± 4.3 pathways (Figure 5A) and 82.7 ± 140.6 genes (Supplementary Figure S3A). The resultant set of SuperPaths indeed enhances the uniqueness *U_S_* as depicted in Figure 5B.

The unification process resulted in relatively small changes in gene count distribution between the original pathways and the resultant SuperPaths (Supplementary Figure S3), suggesting a substantial preservation of gene groupings. Notably, applying pure hierarchical clustering (*T_1_* = *T_2_* = 0.3) resulted in a single very large cluster with 1046 pathways (Figure 3C) and with the same amount of singletons, strongly deviating from the goal of specific pathway annotation for genes (Supplementary Figure S3B). This sub-optimal performance of pure hierarchical clustering is general; any of the examined cases of *T_1_* = *T_2_* (Figure 4B diagonal), shows an *U_s_* + *I_s_* value lower than that for *T_1_* = 0.3 *T_2_* = 0.7.

Each SuperPath is identified by a textual name derived from one of its constituent pathways selected as the most connected pathway (hub) in the SuperPath cluster. For simplicity, the option of *de* *novo* naming was not exercised. Selecting the hub’s name, as opposed to that of the largest pathway, was chosen since this tends to enhance the descriptive value for the entire SuperPath. When more than one pathway has the same maximal number of connections, the larger one is chosen.

### SuperPaths make important gene connections

One of the major implications of the process of SuperPath generation is elucidating new connections among genes. This happens because genes that were not connected via any pre-unification pathway become connected through belonging to the same SuperPath. The unification into SuperPaths is important in two ways: first, it brings, under one roof, pathway information from 12 sources, each individually contributing ∼9000 to ∼5 million instances of gene pairing, for a total of 7.3 million pairs (Supplementary Figure S4). Second, by unifying into SuperPaths, the number of gene pairs is further enhanced, reaching 8.3 million (Supplementary Figure S4).

**Figure 6.**
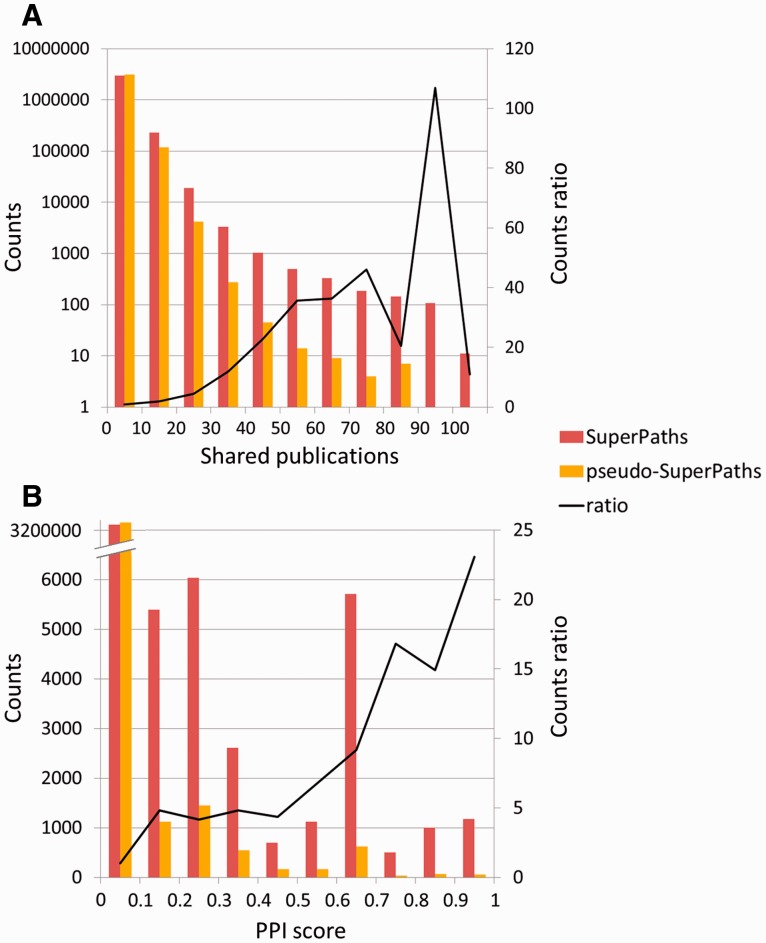
SuperPath-specific gene pairs are informative. **(A)** Shared publications. SuperPath-specific gene pairs are genes connected only by SuperPaths and not by any of the contained pathways. Enrichment of 10–100 is seen in the high abscissa values. The two distributions are significantly different (Kolmogorov–Smirnof *P* < 10^−100^). No random gene pairs with 80–90 publications—this point was treated as having one such publication for computing the ratio. **(B)** Protein–protein interactions. Experimental interaction score from STRING (32) as depicted in GeneCards (12), for SuperPath *versus* random gene pairs as in panel A. The two distributions are significantly different (Kolmogorov–Smirnof *P* < 2.8 × 10^−61^).

**Figure 7.**
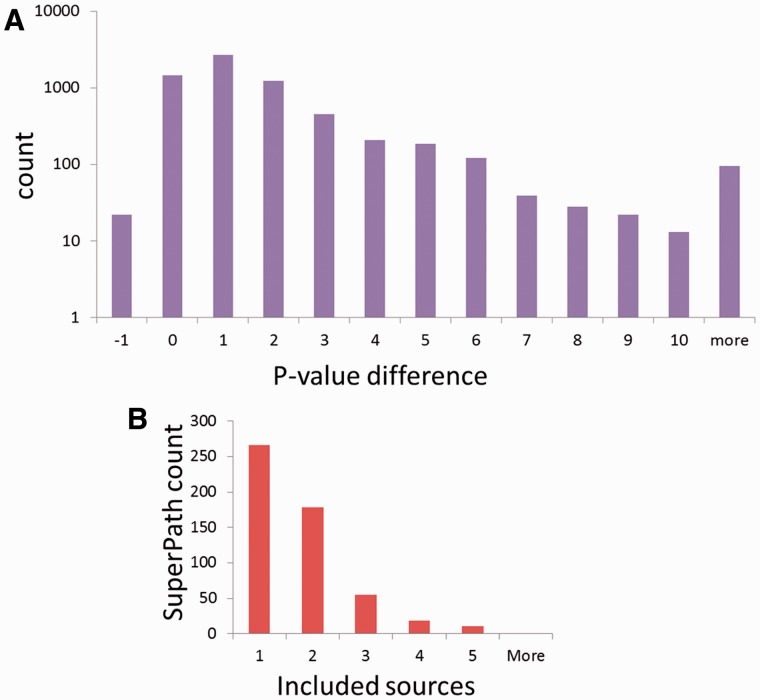
SuperPath integration attributes. **(A)** SuperPaths outperform their constituent pathways in significance scores across 830 differentially expressed genes sets. **(B)** Number of included sources in non-singleton SuperPaths.

To test the significance of the million new gene–gene connections resulting from SuperPath generation, we checked their correlation with two independent measures of gene pairing. First, a comparison was made to publications shared among gene pairs (Figure 6A). We found that for gene pairs appearing in a SuperPath but not in any of its constituent pathways, there is a 4- to 75-fold increase in instances of >20 shared publications when compared with random pairs of genes with pathway annotation. Added gene pairs have significantly more shared publications than those randomly paired. Second, we performed a similar analysis based on protein–protein interaction information. We found that for the SuperPath-implicated gene pairs there was a 4- to 25-fold increase of PPIs with score >0.2 (Figure 6B) when compared with controls. SuperPaths thus provide significant gene partnering information not conveyed by any of their 3215 constituent individual pathways. This may be seen when performing gene set enrichment analysis on 830 differential expression sets and comparing the scores of SuperPaths to that of their constituent pathways, demonstrating that SuperPaths tend to receive more significant scores compared with their constituent pathways average score (Figure 7A).

### SuperPaths in databases

SuperPath information is available both in the GeneCards pathway section (Supplementary Figure S5A) and in PathCards (Supplementary Figure S5B) http://pathcards.genecards.org/, a GeneCards companion database presenting a web card for each SuperPath. PathCards allows the user a view of the pathway network connectivity within a SuparPath, as well as the gene lists of the SuperPath and of each of its constituent pathways. Links to the original pathways are available from the pathway database symbols, placed to the left of pathway names. PathCards has extensive search capacity including finding any SuperPath that contains a search term within its included pathway names, gene symbols and gene descriptions. Multiple search terms are afforded, allowing fine-tuned results. The search results can be expanded to show exactly where in the SuperPath-related text the terms were found. The list of genes in a PathCard utilizes graded coloring to designate the fraction of included pathways containing this gene, providing an assessment of the importance of a gene in a SuperPath. Other features, including gene list sorting and a search tutorial, are under construction. PathCards is updated regularly, together with GeneCards updates. A new version is released 2–3 times a year.

## Discussion

### Pathway source heterogeneity

This study highlights substantial mutual discrepancies among different pathway sources, e.g. with regard to pathway sizes, names and gene contents. The world of human biological pathways consists of many idiosyncratic definitions provided by mostly independent sources that curate publication data and interpret it into sets of genes and their connections. The idiosyncratic view of the different pathway sources is exemplified by the variation in pathway size distribution among sources (Table 1, Figure 2D), where some sources have overrepresentation of large pathways (QIAGEN), while others have mainly small pathways (HumanCyc). In some cases, the large standard deviation in pathway size (Table 1) is easily explained, as exemplified in the case of Reactome, which provides hierarchies of pathways and therefore contains a spectrum of pathway sizes. However, large standard deviations of pathway size are also observed in KEGG and QIAGEN—sources that are not hierarchical by definition. On the other hand, some sources (e.g. HumanCyc, PID and PharmGKB) have very little variation in their pathway sizes, revealing their focus on pathways of particular size. The idiosyncratic view provided by different sources is also evident when examining the genes covered by each source (Figure 2A), where some genes in the gene space are covered by only one source. This causes the unfavorable outcome that when unifying pathways, irrespective of the algorithm chosen, there is a relatively high proportion of single source pathway clusters. In order to account for the drawback of the Jaccard index to cope with large size differences between pathways, we added a preprocessing step to unify pathways that are almost completely included within other pathways (≥0.9 gene content similarity of the smaller pathway), thereby diminishing the barrier of variable pathway size between sources. Previously published isolated instances of intersource discrepancies include the lack of pathway source consensus for the TCA cycle (3) and fatty acid metabolism (2). The authors of both papers stress that each of their pathway sources has only a partial view of the pathway. For the TCA cycle example (3) there is an attempt to provide an optimal TCA cycle pathway by identifying genes that appear in multiple sources, but such manual curation is not feasible for a collection of >3000 biological pathways. In our procedure, 11 relevant pathways from four sources are unified into a SuperPath entitled ‘Citric acid cycle (TCA cycle)’ (Supplementary Figure S5). PathCards enables one to then view which genes are more highly represented within the constituent pathways. Our algorithm thus mimics human intervention, and greatly simplifies the task of finding concurrence within and among pathway sources.

### Pathway unification

Combining several pathway resources has been attempted before, using different approaches. The first method is to simply aggregate all of the pathways in several knowledge bases into one database, without further processing. This approach is taken, for example, by NCBI’s Biosystems with 2496 human pathways from five sources (5) and by PathwayCommons with 1668 pathways from four sources (6). This was also the approach taken by GeneCards prior to the SuperPaths effort described here, where pathways from six sources were shown separately in every GeneCard. While this approach provides centralized portals with easy access to several pathway sets, it does not reveal interpathway relationships and may result in considerable redundancy. The second unification approach, taken by PathJam (7), and HPD (8) provide proteins *versus* pathways tables as search output. This scheme allows useful comparisons as related to specific search terms, but is not leveraged into global analyses of interpathway relations. A third line of action is exemplified by ConsensusPathDB (9), which integrates information from 38 sources, including 26 protein–protein interaction compendia as well as 12 knowledge bases with 4873 pathways. This allows users to observe which interactions are supported by each of the information sources. In turn, hiPathDB (10) integrates protein interactions from four pathway sources (1661 pathways) and creates *ad* *hoc* unified superpathways for a query gene, without globally generating consolidated pathway sets. Finally, a fourth methodology is employed by Pathway Distiller (11), which mines 2462 pathways from six pathway databases, and subsequently unifies them into clusters of several predecided sizes between 5 and 500, using hierarchical clustering. The third method of interaction mapping taken by ConsensusPathDB and HiPathDB differs conceptually from the fourth method of clustering, where the interaction mapping method provides information on the specific commonalities and discrepancies in protein interactions among sources with regard to specific keywords or genes, while the clustering method suggests which of the pathways are similar enough to be considered for the same cluster. Therefore, the third and fourth methods are complementary approaches aimed at utilization of pathway information in different observation levels, where the fourth (clustering) method is independent of user input or search in resultant consolidation. In the study described herein, we pursued a clustering method similar to the fourth methodology taken by Pathway Distiller, namely consolidation of pathways into clusters. However, in contrast to Pathway Distiller, our aim was to create a single coherent unification of biological pathways, which is essential for having a universal set of descriptors when looking at gene–gene relations. The resulting SuperPaths simplify the pathway-related descriptive space of a gene and reduce it 3-fold. Furthermore, the cutoffs in our algorithm are chosen to optimally adjust the criteria of uniqueness and informativeness, thereby reducing the subjective effect of choosing cutoffs arbitrarily or by predetermining the number of clusters.

### SuperPath generation

A crucial element in our SuperPaths generation method is the definition of interpathway relationships. We have opted for the use of gene content, as described by others (11, 33). One could also consider the use of pathway name similarity (11). However, among the 3215 pathways analysed here, only 79 names were shared by more than one pathway, implying that the efficacy of such an approach would have been rather limited. Further, Table 2 and Supplementary Figure S2 indicate a relatively weak concordance between pathway names and their gene content. Specifically among 79 name-identical pathway groups 52 remained incompletely unified, again suggesting a limited usefulness for unifying based on pathway names. Many resources, including ConsensusPathDB (9) facilitate the option of finding pathways based on keywords in the name. Name sharing is thus a relatively trivial task to overcome when trying to find similar pathways. The more challenging goal is finding pathways that are similar in the biological process that they convey.

In this article we treated pathways as sets of genes, using gene content as a comparative measure and omitting topology and small molecule information. This approach was previously advocated as a means of reducing the complexity of pathway comparisons greatly (34). Further, most sources used in this study provide only the gene set information, hence topology information was unavailable. Finally, the high concordance between significance of pathway alignment and Jaccard coefficients ≥0.3 (*P* < 10^−^^52^) indicates that the Jaccard coefficient is a good approximation of the more elaborate pathway alignment procedure (25).

### SuperPath utility

A central aim of pathway source unification is enhancing the inference of gene-to-gene relations needed for pathway enrichment scrutiny (32, 35–40). To this end, we developed an algorithm for pathway clustering so as to optimize this inference and at the same time minimize redundancy.

Extending pathways into SuperPaths affords two major advantages. The first is augmenting the gene grouping used for such inference. Indeed, SuperPaths have slightly larger sizes than the original pathways, as evident by the SuperPath size distribution (Figure 2D). Nevertheless, comparing SuperPaths to pseudo-SuperPaths of the same size and quantity clearly show that the increase in size does not account for the addition of true positive gene connections, as evident by the higher PPIs and larger counts of shared publications for SuperPath gene pairs (Figure 6). Subsequently, it is not surprising that SuperPaths outperform their average pathway constituent’s enrichment analysis scores (Figure 7A). SuperPaths are currently used in two GeneCards-related novel tools, VarElect http://varelect.genecards.org/ and GeneAnalytics http://geneana lytics.genecards.org/. A second advantage of SuperPaths is in the reduction of redundancy, since they provide a smaller, unified pathway set, and thus diminish the necessary statistical correction for multiple testing. We note that ConsensusPathDB (9) also provides intersource integrated view of interactions. However, gene set analysis in ConsensusPathDB is only allowed for pathways as defined by the original sources. Finally, a third advantage of SuperPaths is their ability to rank genes within a biological mechanism via the multiplicity of constituent pathways within which a gene appears. This can be used not only to gain better functional insight but also to help eliminate suspected false-positive genes appearing in a minority of the pathway versions. A capacity to view such gene ranking is available within the PathCards database.

## Limitations of SuperPaths

The SuperPaths generation procedure appears incomplete, as about a half of all SuperPaths are ‘singleton SuperPath’ (labelled accordingly in PathCards), having only one constituent pathway. This is an outcome of the specific cutoff parameters used. However, this provides a useful indication to the user that a singleton pathway is distinct, differing greatly in its constituent genes from any other pathway.

This SuperPath generation process is intended to reduce redundancies and inconsistencies found when analysing the unified pathways. Although SuperPaths increase uniqueness as compared with the original pathway set (Figure 5B), some redundancy and inconsistency still remain within SuperPaths. There are cases of pathways with similar names, which do not get unified into the same SuperPath. This happens because they have not met the unification criteria employed. We also note similarity in name does not always indicate similarity in gene content (Figure 2B and C, Supplementary Figure S2B), and such events are faithfully conveyed to the user.

A clarifying example is that of the 40 pathways whose names include the string ‘apoptosis’. The final post-unification list has 10 SuperPaths whose name includes ‘apoptosis’. This obviously provides the user with a greatly simplified view of the apoptosis world. Yet, at the same time the outcome is replete with instances of two name-similar pathways being included in different SuperPaths. Employing a more stringent algorithm would result in over-clustering, which would in turn reduce informativeness (see Figure 3C).

In parallel, there are pathways with overlapping functions that are not consolidated into one SuperPath. For example, the pathway ‘integrated breast cancer pathway’ does not unify with the pathways ‘DNA repair’ and ‘DNA damage response pathway’, despite the strong functional relation of breast cancer with DNA damage and repair (41). This is because the relevant gene content similarity in the original pathway sources is small, respectively, *J* = 0.03 and 0.13. The need to view information on pathways with low pairwise similarity is addressed in Supplementary Figure S6, and is available as a text file upon request.

Finally, when looking at the number of contributing sources per SuperPath (Figure 7B), it is evident that the majority of SuperPaths are comprised by either one or two sources, and no SuperPaths includes more than five. Although this integration limitation is evident, it mainly arises from the inherent biases in gene coverage for the different information sources (Figure 2A).

### PathCards

Biological pathway information has traditionally been a central facet of GeneCards, the database of human genes (12, 42, 43). In previous versions, pathways were presented separately for each of the pathway sources, and it was difficult for users to relate the separate lists to each other. As a result of the consolidation into SuperPaths described herein, this problem has been effectively addressed. Thus, in every GeneCard, a table portrays all of a gene’s SuperPaths, each with its constituent pathways, with links to the original sources (Supplementary Figure S5A).

GeneCards is gene-centric and inherently does not present (Super) pathway-centric annotations. We therefore developed PathCards http://pathcards.genecards.org/, a database that encompasses and displays such information in greater detail. PathCards has a page for every SuperPath, showing the connectivity of its included pathways, as well as gene lists for the SuperPath and its pathways. For every SuperPath, we also show a STRING gene interaction network (32) for the entire gamut of constituent genes, providing perspective on topological relationships within the SuperPath.

## Supplementary Data

Supplementary data are available at *Database* Online.
